# PRECISION IN HEALTH CARE FOR DEMENTIA: DO CARE PARTNERS KNOW WHAT THEY DON’T KNOW?

**DOI:** 10.1093/geroni/igz038.2208

**Published:** 2019-11-08

**Authors:** Tatiana Sadak, Emily Ishado, Soo Borson

**Affiliations:** University of Washington, Seattle, Washington, United States

## Abstract

Clinicians rely on care partners to provide health care at home for people with dementia, who typically have multiple chronic conditions in addition to progressive cognitive decline. We examined the accuracy of care partners’ knowledge of care recipients’ medical conditions and medications, using a benchmark of ≥ 80% match. Of 100 care partners of people with dementia who were recently hospitalized for a major medical illness, nearly all rated their knowledge as high, but about half did not correctly identify care recipients’ medical conditions or know medications, and one fourth did not understand the purpose for which medications were given. A key predictor of poor objective knowledge was care partners’ cognitive status. These findings highlight the importance of objective assessment of care partner knowledge and skills by clinicians who provide health care and advance care planning for people with dementia.

